# Emerging SARS-CoV-2 variants of concern and potential intervention approaches

**DOI:** 10.1186/s13054-021-03662-x

**Published:** 2021-07-12

**Authors:** Jasmin Khateeb, Yuchong Li, Haibo Zhang

**Affiliations:** 1 Keenan Research Centre for Biomedical Science, St. Michael’s Hospital, Unity Health Toronto, Room 619, LKSKI, 30 Bond Street, Toronto, ON M5B1W8 Canada; 2 Department of Internal Medicine D, Rambam Health Care Campus, Haifa, Israel; 3 The State Key Laboratory of Respiratory Disease, Guangzhou Institute of Respiratory Disease, The First Affiliated Hospital of Guangzhou Medical University, Guangzhou, Guangdong China; 4 Departments of Anaesthesia and Physiology, Interdepartmental Division of Critical Care Medicine, University of Toronto, Toronto, ON Canada

**Keywords:** Transmissibility, Viral virulence, B.1.1.7, B.1.351, P.1. B.1617.1

## Abstract

The major variant of concerns (VOCs) have shared mutations in severe acute respiratory syndrome coronavirus 2 (SARS-CoV-2) spike proteins, mostly on the S1 unit and resulted in higher transmissibility rate and affect viral virulence and clinical outcome. The spike protein mutations and other non-structural protein mutations in the VOCs may lead to escape approved vaccinations in certain extend. We will discuss these VOC mutations and discuss the need for combination therapeutic strategies targeting viral cycle and immune host responses.

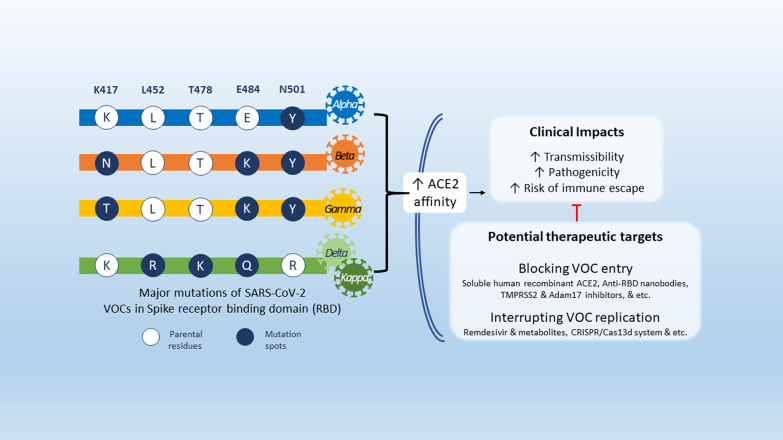

## Background

The surge of new severe acute respiratory syndrome coronavirus 2 (SARS-CoV-2) variants of concern (VOC) poses a major threat on international public health as the rapid change of the SARS-CoV-2 genome may alter viral phenotypes such as virulence, transmissibility, and ability to evade host response giving rise to greater challenge to diagnostic and clinical management [1, 2]. Yet, it is unclear whether the approved vaccines are effective against the VOCs. This article will summarize the main emerging VOCs and their potential epidemiological impact aiming at promoting development of drug therapy and effective vaccines and formulation of public health strategies.

## SARS-CoV-2 genome and mutations

SARS-CoV-2 is a positive-sense single-stranded RNA virus whose genome is of a low stability thus is more prone for mutation accumulation, with approximately 9.8 × 10^−4^ substitutions/site yearly [3–7]. The architecture of SARS-CoV-2 is made of two groups of proteins: structural proteins (SP) and non-structural proteins (NSP). SPs are encoded by 4 genes, including E (envelop), M (membrane), S (spike) and N (nucleocapsid) genes [8]. NSPs are mostly enzymes or functional proteins that play a role in viral replication and methylation and may induce host responses to infection. These genes are encoded in several groups, namely ORF1a (NSP1-11), ORF1b (NSP12-16), ORF3a, ORF6, ORF7a, ORF7b, ORF8 and ORF10.

A variant can be as simply as a virus bearing a deviant mutation or complicated combinations of deviations leading to significant phenotypical alteration from original genome. Although by the beginning of May 2021, there has been reported more than 1.4 million sequences and among them 3913 major representative variants genomes that have been identified and included in the global SARS-CoV-2 sequence database operated by Global Initiative on Sharing Avian Influenza Data (GISAID) [9], not all genetic mutations lead to variation in major proteins and/or alter virus infectivity. The spike gene mutations account for most of the clinically influential VOCs while the ORF1a frame of the genome serves as a key region for NSP mutations.

We will focus our discussion here on the VOCs that have major global health impacts since the 4^th^ quarter of the year 2020, including Alpha variant (B.1.1.7), Beta (B.1.351), Gamma (P.1) and Kappa and Delta (B.1.617.1 and B.1.617.2) (Table 1).

**Table 1:** Molecular and clinical characteristics of SARS-CoV-2 variants of concern

Variant of concern		Alpha B.1.1.7	Beta B.1.351	Gamma P.1	Delta and Kappa* B.1.617.2 B.1.617.1
Epidemiology	First Identified	September. 2020, UK	October. 2020, South Africa	December. 2020, Japan & Brazil	December. 2020, India
	Global frequency**	48%	7%	7%	14%
	Major geographic distribution	Worldwide	South Africa	South America	Asia
Predominant mutations	Spike RBD mutations	N501Y	K417N, E484K, N501Y	K417T, E484K, N501Y	L452R, E484Q, T478K (Delta)
	Spike non- RBD mutations	D614G, P681H	D614G	D614G	D614G, P681R
Clinical considerations ***	Transmissibility	↑ ****	↑?	↑?	↑?
	Virulence	**↑?**	**↑?**	↑?	↑?
	Host Immune response	↓	↓	↓?	↓?
	Diagnostic tools	↔	↔	↔	↔
Therapeutic considerations***	**Vaccinations’ effectivity**				
	mRNA- based	↔	↓	↔	?
	Adenovirus-based	↓	↓	↓	?
	Recombinant protein-based vaccines	↓	↓↓	?	?
	Inactivated virus-based	↔	↓	↔	?
	**Potential therapeutic strategies**				
	S1 RBD targeted therapeutics: Soluble human recombinant ACE2, anti-RBD nanobodies Endosomal formation interruption: TMPRSS2 inhibitors (e.g., Camostat), ADAM17 inhibitors, Viral replication-oriented therapies: RdRp inhibitors (e.g. Remdesivir, GS-441524), Cas13d-based PAC-MAN				

*Delta and Kappa are derived from the emerging lineage B.1.617; **Last updated June 6^th^, 2021; ***All data is suggestive according to in vitro experiments unless mentioned otherwise; ****Clinical data showed higher transmissibility rate of 35–45% especially in younger group ages and children.

### Spike mutations

Spike protein mediates the virus attachment to human cell surface angiotensin converting enzyme 2 (ACE2) receptor, thus facilitating viral entry during infection [10–12]. It is split into two subunits, S1 and S2. The S1 unit possess the receptor-binding domain (RBD) which can directly bind to ACE2 receptor and is also the dominant target of neutralizing antibodies (Ab) against SARS-CoV-2. S1 is thus considered a hotspot for mutations that may have high clinical relevance in terms of virulence, transmissibility, and host immune evasion [13–16] (Table 2).

**Table 2:** Major spike mutations

Mutating residues	Mutation	Region	Pathophysiology		Clinical Impact expectations		Major variants			
			ACE2 affinity	Immune escape^#^	Transmissibility	Virulence	Alpha B.1.1.7	Beta B.1.351	Gamma P.1	Delta &Kappa B.1.617.2/1
K417	K417N	RBD	↑	↑	↑	↑		√		
	K417T		↑	↑	↑	↑?			√	
L452	L452R		↑	↑	↑	↑				√
T478	T478K		↑	↑	↑	↑?				√ (Delta)
E484	E484K		↑	↑	↑?	↑?	√ (partially)	√	√	
	E484Q		↑	↑	↑?	↑?				√
N501	N501Y		↑	↑	↑	↑	√	√	√	
D614	D614G	non-RBD	↑	↑	↑	↔	√	√	√	√
P681	P681H	S1/S2 Furin cleavage site	↔	↑?	↑	↑?	√			
	P681R		↔	↑?	↑	↑?				√

^#^Both host and vaccine-induced immunity. Abbreviations: RBD, receptor binding domain

The Alpha variant has an N501Y mutation: at the 501 residue, N asparagine has been replaced with Y tyrosine, as well as K417N—lysine K replaced with asparagine N [9]. An emerging variant derived from B.1.1.7 also carries E484K mutation—glutamic acid E replaced with lysine K [9]. Both Beta and Gamma variants have more substitutions other than N501Y [9]. The Beta variant has E484K, while the Gamma variant has the E484K and the K417T mutations [9]. The latest major variants, Delta and Kappa, sharing two mutations E484Q (glutamic acid E substituted by glutamine Q) and L452R (leucine L altered by arginine R) were identified in India’s second COVID-19 wave. Other than the two mutations above, Delta also harbours a unique mutation, T478K (threonine T replaced by lysine K) [9].

The S1 mutations significantly increases the binding affinity to ACE2 while showing lower affinity to neutralizing antibodies [17–21], suggesting a possible explanation for their occurring higher transmissibility and virulence [22, 23].

Another mutation at non-RBD sites, named D614G, is the most spreading mutation carried by over 99% of prevalent variants since early 2020 [23, 24]. Such mutation does not change the binding affinity to ACE2 or neutralizing Abs for the virion, yet it may increase spike density by preserving the integrity of spike and avoiding S1 shedding [25]. With more functional spikes available, D614G variants are armed with increased infectivity and hence increased replication in vitro while earlier transmission in vivo [23, 25, 26]. Recently, increasing deletions are observed in the neutralizing Ab-recognizing domain, namely recurrent deletion regions (RDRs), in the N-terminus of S1 subunit [27]. Deletions in RDRs wipe out the epitopes, and eventually aiding the virus evading host’s immune supervision and potentially defecting certain neutralizing Abs or vaccines. A majority of Alpha derived variants (ΔRDR1, S: ΔHV 69–70, & ΔRDR2, S: ΔY144), Beta derived variants (ΔRDR4, S: ΔLAL 242–244) and B.1.36 (ΔRDR3, S: ΔI210) carry this kind of mutation [27].

### NSP mutations

Two mutation hot-spots, NSP1 of ORF1a/ORF1ab, and ORF8, have been found related to the virulence and transmissibility. NSP1 is a key protein to antagonize type I interferon induction in the host and benefit the replication of the virus itself [28, 29]. ORF8 is known as an immune-evasive protein that downregulates major histocompatibility complex class I (MHC-I) in host cells [30, 31]. Recently, the Alpha variant, identified from a single immunocompromised individual, was shown to contains a premature stop codon at position 27 of ORF8[32].

Variants with partial deletion of NSP1 and ORF8 have been identified (e.g., the NSP1: Δ500-532 variant in Sichuan, China, and the ORF8: Δ382 variant in Singapore) [29, 31]. Despite that truncated NSP1 and ORF8 both contribute to milder infections [29–31] and account for less than 5% of infections worldwide, they have become the major variants in Africa since late 2020[9].

## Potential clinical impacts of SARS-CoV-2 VOCs

### Increased transmissibility and viral virulence

It was shown that S-protein mutation D614G may impact SARS-CoV-2 transmissibility rate due to higher affinity for olfactory epithelium and it was shown to have higher transmissibility in animal models [33, 34]. It was also shown that it has a higher virion stability and was shown to be more resistant to proteolytic cleave as well as higher viral titer in upper airways [35, 36] suggesting that it may potentially affect virus transmissibility and virulence. Yet, it showed increased susceptibility for neutralizing antibodies and no difference in clinical severity nor hospitalization outcomes and mortality was observed [37, 38].

Evidence suggest that the VOCs Alpha and Beta increased transmissibility rate at ~ 50% especially in younger group ages and children [39, 40]. Alpha variant was shown to increase hospitalizations and mortality that may be attributed to their escape from neutralizing Abs due to their RBD mutations [41].

The Epsilon variant (B.1.427/B.1.429, California variants) increased transmissibility up to 24% with higher viral shedding, which is attributed to the of L452R spike mutation that was shown to stabilize spike-ACE2 receptor interaction [42, 43].

Although it is also suggested that other variants such as Gamma, Epsilon variants and recent Iota variants (B.1526, New York variant) may also have increased virulence due to spike mutations that increase affinity to ACE2, there is still no data available regarding viral virulence.

### Decreased diagnostic sensitivity

The new VOCs can reduce the detection sensitivity of RT-PCR based diagnostic tools especially when mutations occur in locations where probes and primers may bind [44]. Reports suggest that 79% of the primer binding sites used in the RT-PCR assay are already mutated in at least one genome with the highest significance of the GGG → AAC substitution [45]. Recent analysis which mapped primers or probes binding sites showed a cumulative variants frequency of ≥ 1% in the global SARS-CoV-2 genomes [46]. The Alpha lineage was shown to have higher false-negative results when using specific commercial kits directed to the spike (S) gene but not when using standard protocols such as Berlin-Cherite protocol since it does not involve the S protein-encoding gene as target [47]. Another concern is a variant detected in France of a S deletion (ΔH69-V70) which has shown to be associated with S-gene target gene detection failure in three-target RT-PCR [48]. Several reports have targeted mutations in different open reading frames (ORFs) especially ORF8 position which was found in some isolates from Mexico, Belize and Guatemala as potentially leading to epitope loss and reduced sensitivity for serological testing [49–51].

On the other hand, other studies showed that although mismatches in the primer/probes binding regions of SARS-CoV-2 diagnostic assays can be detected in different SARS-CoV-2 variants, they were tolerated and did not result in reduced assay performance and false-negative results [52, 53]. Moreover, according to bioinformatic analysis performed, the known variability occurring in the SARS-CoV-2 population have minimal or no effect on the sensitivity existing diagnostic tools for viral detection [54, 55].

Still, the continuous emergence of SARS-CoV-2 variants and possible mismatches highlight the importance of global molecular surveillance and designing diagnostic strategies such as combining diagnostic methods during future outbreaks or perform assays that target two or more positions in highly conserved regions of the viral genome to promote higher specificity and sensitivity results as well as developing highly specific diagnostic tools using CRISPR [56, 57].

### Potential influence on vaccination

Currently, all vaccines are based on introducing spike protein, which is the major superficial virulence of SARS-CoV-2, using the reference genome isolates early in the pandemic. As there is no sufficient evidence to support the effect of vaccines against Delta and Kappa variants, we’ll focus on the Alpha, Beta and Kappa variants.

#### mRNA Vaccines

Two major mRNA-based anti-SARS-CoV-2 vaccines have been approved: BNT162b2 (Pfizer-BioNTech) and mRNA-1273 (Moderna). Studies suggest that BNT162b2 vaccines were able to stimulate the recipients to generate capable antibodies to neutralize Alpha and Gamma variants yet being significantly less protective against Beta variant [41, 58, 59] mRNA-1273 was shown to enhance sufficient neutralizing ability against Alpha variant yet lower reciprocal titer against Beta variant [41, 60, 61].

#### Adenovirus-based vaccines

There are 4 adenovirus-based vaccines that have been authorized for general or emergency use. Among of which, Ad26.COV2.S is a recombinant, replication-incompetent adenovirus serotype 26 (Ad26) vector encoding a full-length and stabilized SARS-CoV-2 spike protein (Janssen) was shown to have reduced efficacies to Beta variant (64%) and Gamma dominant Latin America variant (61%), compared to the U.S. (72%) where Alpha is the dominant strain [62]. ChAdOx1 nCoV-19 (Oxford) is a chimpanzee adenovirus-vectored vaccine expressing the SARS-CoV-2 spike protein. Recent studies revealed that the efficacy of ChAdOx1 nCoV-19 was 74.6% against Alpha but as low as 10.4% against Beta [63, 64]. Gam-COVID-Vac (Ad26 and Ad5) is also claimed protective to the global VOCs, yet the clinical trial result has not yet been publicized [65]. An ongoing clinical trial on the combination of ChAdOx1 nCoV-19 and Gam-COVID-Vac (Russia), which is a heterologous COVID-19 vaccine consisting of two components, a recombinant adenovirus type 26 (rAd26) vector and a recombinant adenovirus type 5 (rAd5) vector, both carrying the gene for SARS-CoV-2 spike glycoprotein. There is no data regarding its efficacy on VOCs [66].

#### Subunit vaccines

NVX-CoV2373 (Novavax) contains a full-length, prefusion spike protein, and shows an 86.3% efficacy against Alpha, yet 48.6% against Beta [67]. However, none of the recombinant protein-based vaccines have yet to be approved for general use.

#### Inactivated virus-based vaccines

Three inactivated virus- based vaccines have been approved so far and have been widely used in China, India and Brazil. A recent in vitro study suggests that antisera elicited by BBIBP-CorV vaccine (Sinopharm) are able to neutralize the Beta variant in a differentially weaker level compared to the wildtype strain and the D614G variant [68]. A recent serological study has shown that BBV152 (Bharat Biotech International Limited) vaccinated human serum is able to neutralize the Alpha variant [69]. Preliminary data from a study conducted in Sao Paulo, Brazil indicate that the most widely vaccinated vaccine, CoronaVac (Sinovac Biotech), is effective against Gamma variant [70]. The same research facility claimed the vaccine also ‘works well’ against the Alpha and Gamma variants [71].

To conclude, it appears that Beta is most likely variant to affect the approved vaccines efficiency while Alpha and Gamma variants do not. These results suggest that a new vaccine might be required specifically to target Beta variant. Many strategies are currently under development to cope with Beta variant challenge such as booster vaccines [72].

## Potential therapeutics for VOCs

### S1 RBD targeted therapy

Whether specifically targeting spike proteins using small peptide-based therapies or using single-domains neutralizing antibodies against any of those targets, these therapeutic strategies efficiency may be compromised by the emergence of SARS-CoV-2 variants especially those possessing spike proteins and RBD mutations that increase affinity to ACE2 such as Alpha, and Iota variant, by potentially escaping neutralizing antibodies and competing with those agents for the same binding targets [73–75]. In order to avoid antibody escape, strategies to combine different neutralizing antibody cocktail have been suggested as a therapeutic approach against the emerging variants [76]. Other treatments such as anti-RBD nanobodies isolated from llamas were shown to neutralize RBD variants suggesting they might be a promising tool against new SARS-CoV-2 VOCs as well [77, 78].

Different engineered variants of human recombinant soluble ACE2 (hrACE2), were reported to significantly inhibit SARS-CoV-2 infection in vitro and causing sustained viral entry blockade upon engagement of hrACE2 with the RBD in SARS-CoV-2 S protein with high affinity [79–81]. This is a potentially powerful treatment against SARS-CoV-2 VOCs as it can exploit the increase S-protein host receptor-binding affinity caused by S-mutations, toward increasing S-protein affinity to hrACE2. Moreover, no mutations that limit receptor-binding affinity were discovered as this will decrease affinity to native ACE2 receptor and may likely to attenuate virulence [82], suggesting that viral escape from hrACE is very unlikely.

### Interruption of endosomal formation

Targeting endosomal formation of SARS-CoV-2 to block entry to host cells such as antimalarial drugs and macrolides, and us of drugs targeting host cell transmembrane protease serine 2 (TMPRSS2) such as Camostat [83–85] or A disintegrin and metalloprotease 17 (ADAM17) inhibitors [86].

### Interruption of SARS-CoV-2 VOC genome

Promising antiviral drugs such as the FDA-approved Remdesivir and its metabolites, Ribaverin and Galidesivir have been shown to inhibit viral replication in vitro and in vivo studies due to their effect on inhibiting RNA dependent RNA polymerase (RdRp) [87, 88]. The discovery of RdRp hotspot mutations in SARS-CoV-2, found mostly in European strains may lead to drug-resistance of to RdRp inhibitors in a similar mechanism found in Influenza and Hepatitis C [89–91]. However, it has been shown currently that those variants have minimal impact for pre-existing resistance to Remdesivir.

Another potential approach is Prophylactic Antiviral CRISPR in Human Cells (PAC-MAN), which is a Cas13d-based strategy that target reserved regions such as nucleocapsid protein and RdRp in SARS-CoV-2 viral genome and may serve as pan-coronavirus strategy for any future coronaviruses and variant that may emerge [92].

## Conclusion

Emerging VOCs have the potential to effect clinical and global health outcomes, emphasizing the necessity for genomically tailored therapeutic approach in the future therefore we suggest that a combination strategy targeting different components in viral cycle and immune host response may be critical but overlooked in the combat against SARS-CoV-2 VOCs.

## Data Availability

Not applicable.

## Abbreviations

Ab: Antibodies
ACE2: Angiotensin Converting Enzyme 2
ADAM17: A disintegrin and metalloprotease 17
E: Envelope
GISAID: Global Initiative on Sharing Avian Influenza Data
hrACE2: Human Recombinant Soluble Angiotensin Converting Enzyme 2
M: Membrane
MHC-I: Major Histocompatibility Complex Class I
N: Nucleocapsid
NSP: Non-Structural Proteins
ORF: Open Reading Frames
PAC-MAN: Prophylactic Antiviral CRISPR in Human Cells
RBD: Receptor-Binding Domain
RDR: Recurrent Deletion Regions
RdRp: RNA dependent RNA polymerase
S: Spike
SARS-CoV-2: Severe Acute Respiratory Syndrome Coronavirus 2
SP: Structural Proteins
TMPRSS2: Transmembrane Protease Serine 2
VOC: Variant Of Concern
